# Virulence genotype and phenotype of two clinical isolates of *Arcobacter butzleri* obtained from patients with different pathologies

**DOI:** 10.1007/s00203-023-03709-3

**Published:** 2023-11-04

**Authors:** Itsaso Baztarrika, Adrián Salazar-Sánchez, Silvia Hernaez Crespo, José Israel López Mirones, Andrés Canut, Rodrigo Alonso, Ilargi Martínez-Ballesteros, Irati Martinez-Malaxetxebarria

**Affiliations:** 1 MikroIker Research Group, Department of Immunology, Microbiology and Parasitology, Faculty of Pharmacy, University of the Basque Country UPV/EHU, Paseo de la Universidad 7, 01006 Vitoria-d Gasteiz, Spain; 2 Bioaraba, Microbiology, Infectious Diseases, Antimicrobial Agents and Gene Therapy, 01006 Vitoria-Gasteiz, Spain

**Keywords:** *Arcobacter butzleri*, Caco-2 infection, Comparative genomics, Antimicrobial resistance, Virulence

## Abstract

The surge in human arcobacteriosis has increased interest in determining the mechanisms involved in the pathogenesis of *Arcobacter butzleri*. Here, genomic analyses and in vitro Caco-2 infection, motility, urease and antimicrobial susceptibility testing (AST) assays were used to characterise the virulence and antimicrobial resistance (AMR) determinants of strains HC-1, isolated from a patient with travellers’ diarrhoea, and HC-2, isolated from another with pruritus. AMR determinants conferring resistance to tetracycline (*tetO*, present in both genomes) and to ampicillin and amoxicillin–clavulanic acid (*bla3*, present in HC-2) were identified. The same determinants associated with flagellum, chemotaxis, adhesion and invasion were detected in both, but HC-1 lacked eight flagellar genes. The urease cluster was only present in HC-1. Motility and urease tests confirmed the genetic differences between strains, but no genetic marker related to the inability of HC-2 to adhere and invade was identified. This inability could be conditioning the patient’s pathology.

**Supplementary Information:**

The online version contains supplementary material available at 10.1007/s00203-023-03709-3.

## Introduction

*Arcobacter butzleri*, along with some other species of the genus, has been considered a serious hazard to human health since 2002 (ICMSFS 2002). The increase over the last few years in reported cases of human infection with *A. butzleri* has led to a growing interest in this species (Jiménez-Guerra et al. 2020; Ruiz de Alegría Puig et al. 2023; Simaluiza et al. 2021). In fact, *A. butzleri* turns out to be the species of the genus that most frequently causes human infection (Chieffi et al. 2020). However, this emerging enteropathogen usually goes unnoticed in hospital microbiology laboratories and its incidence may be underestimated (Ruiz de Alegría Puig et al. 2023). The symptomatology described in the different clinical cases is very diverse (Ramees et al. 2017), so it would be very important to further study the pathogenic potential of *Arcobacter* by characterising clinical strains*.* In particular, *A. butzleri* has been primarily associated with enteritis, being the main symptom a persistent diarrhoea (Chieffi et al. 2020). In addition, this bacterium has also been linked to cases of bacteraemia (Arguello et al. 2015) and peritonitis (Yap et al. 2013) in immunocompromised patients, and to asymptomatic infections (Jiménez-Guerra et al. 2020). Despite limited knowledge on the mechanisms of pathogenesis of this species, it is known that *A. butzleri* has a great ability to adhere to and invade different cell lines (Bücker et al. 2009; Fallas-Padilla et al. 2014; Ferreira et al. 2014; Gugliandolo et al. 2008; Ho et al. 2007; Karadas et al. 2013, 2016; Levican et al. 2013), including human intestinal cells (Buzzanca et al. 2021; Ferreira et al. 2014; Ho et al. 2007; Karadas et al. 2013). This virulent capacity causes a dysfunction of the epithelial barrier, which is related to the onset of severe diarrhoea (Bücker et al. 2009). However, deeper studies are needed to increase the knowledge of the virulent capability of this microorganism (Ferreira et al. 2014; Karadas et al. 2016; Levican et al. 2013).

Typically, the pathogenic potential of *Arcobacter* spp. has been determined by the detection of ten virulence markers: *cadF* and *cj1349*, which encode fibronectin binding proteins that promote the binding of bacteria to intestinal cells (Dasti et al. 2010; Konkel et al. 2005); *ciaB* (*Campylobacter* invasive antigen B), which contributes to host cell invasion through a secretion system (Konkel et al. 1999); *hecA*, involved in attachment, aggregation and epidermal cell killing (Rojas et al. 2002); *hecB* and *tlyA*, coding for a haemolysin activation protein and a haemolysin, respectively (Miller et al. 2007; Wren et al. 1998); the phospholipase encoding gene *pldA*, also associated with erythrocyte lysis (Istivan et al. 2008); *mviN*, coding for a protein essential for peptidoglycan biosynthesis (Inoue et al. 2008); and *irgA* and *iroE*, that code for functional components for iron acquisition and are required for establishing and maintaining infection (Goldberg et al. 1990). However, whole genome sequencing (WGS) has proven to be a useful technique to further expand knowledge about *Arcobacter,* and several studies have focused on using it to provide more information (Buzzanca et al 2021; Fanelli et al. 2019; Isidro et al. 2020; Ma et al. 2022; Miller et al. 2007; Müller et al. 2020a, b). It should be noted that although the first genome of *A. butzleri* was sequenced and analysed several years ago (Miller et al. 2007), until 2020, no comprehensive genome-scale comparative analysis of *A. butzleri* has been performed (Buzzanca et al. 2021; Isidro et al. 2020; Müller et al. 2020a, b). These latest studies corroborate the high diversity and plasticity of the genome of this emerging human pathogen, highlighting its pathogenic potential and adaptive capacity.

Knowing the importance of further understanding of the pathogenic characteristics of *A. butzleri*, this study aims to characterise the virulence potential of two strains of *A. butzleri* isolated from two patients, one diagnosed with diarrhoea and the other with pruritus, through the combination of genomic data analysis and phenotypic techniques.

## Materials and methods

### Clinical characteristics of the patients and microbiological examination

Each patient, a 28-year-old woman and 57-year-old man, presented different symptoms. The woman, who had travelled to Thailand a year ago, was suffering from watery diarrhoea and low-grade fever for days prior to the episode. The man suffered from prostate cancer and was diagnosed with pruritus.

In both cases, stool samples were taken and tested for pathogenic bacteria, Rotavirus, Norovirus, Astrovirus, Adenovirus and eggs or/and cysts from parasites at the Microbiology Service of the University Hospital of Araba (HUA) in 2017. The media and techniques used were Cefsulodin-Irgasan-Novobiocin (CIN), Hecktoen, Campylosel and Salmonella Shigella (SS) agars (bioMerieux, Marcy-l'Étoile, France) for bacteria; Rota + Adeno + Astro + Norovirus 1/2 combo card (CerTest Biotec, Zaragoza, Spain) for viruses; and Crypto + Giardia + Entamoeba combo card (CerTest Biotec, Zaragoza, Spain) and Mini PARASEP (Apacor, Berkshire, United Kingdom) for parasites.

The isolated bacteria were identified by matrix-assisted laser desorption/ionisation time-of-flight mass spectrometry (MALDI-TOF MS, Microflex LT, Bruker Daltonics, Bremen, Germany). For further analysis, isolates were inoculated into brain heart infusion (BHI) broth (Oxoid, Basingstoke, UK), and stored at − 80 ℃ as suspensions in BHI with 25% glycerol.

The woman was treated with erythromycin and progressed well. The man was treated with cetirizine alone and progressed well, without needing antibiotic therapy.

### Bacterial strains and growth conditions

Two *A. butzleri* isolates from human faeces, HC-1 and HC-2, were characterised here by means of genomic and in vitro assays. In the Caco-2 cell line infection assays, strains *Salmonella enterica* serovar Typhimurium LT2 CECT 722 (Spanish Culture Cell Type), *Escherichia coli* DH5α NCCB 2955 (Netherlands Culture Collection of Bacteria), and *A. butzleri* RM4018 (CCUG 30485, Culture Collection University of Gothenburg) were also included; *S.* Typhimurium LT2 as positive control for the adhesion and invasion*, E. coli* DH5α as positive control for adhesion and negative for invasion, and *A. butzleri* RM4018 as reference. This latter strain was also included as positive control in the motility and urease activity tests. In addition, *E. coli* DH5α strain was included as negative control in the urease test.

*Arcobacter* strains were routinely grown at 30 °C for 12–16 h in BHI broth (Oxoid, Basingstoke, UK) or for 24–48 h on Columbia agar base plates (Oxoid, Basingstoke, UK) supplemented with 5% defibrinated sheep blood (Liofilchem, Roseto degli Abruzzi, Teramo, Italy), under aerobic conditions. *S.* Typhimurium LT2 and *E. coli* DH5α were routinely grown at 37 °C for 12–16 h in BHI broth (Oxoid, Basingstoke, UK) or on Muller–Hinton (MH) agar (Oxoid, Basingstoke, UK), aerobically. Shaking (150 rpm) was applied when necessary.

### Antimicrobial susceptibility testing

As part of the microbiological diagnostic procedure, minimum inhibitory concentrations (MIC) for ciprofloxacin, erythromycin, tetracycline, amoxicillin–clavulanic acid, ampicillin and gentamicin were determined at the HUA. The gradient strip diffusion method (MIC Test Strip; Liofilchem, Roseto degli Abruzzi, Teramo, Italy) was used for this purpose and the strains were classified as susceptible or resistant according to the breakpoints proposed by the European Committee on Antimicrobial Susceptibility Testing (EUCAST) for *Campylobacter jejuni/col*i (ciprofloxacin, erythromycin and tetracycline) and for *Enterobacterales* (amoxicillin–clavulanic acid, ampicillin and gentamicin).

### Caco-2 cell line infection assays

Cell adhesion and invasion ability of both strains was studied by infecting monolayers of the human colorectal adenocarcinoma cell line Caco-2 (ATCC^®^ HTB-37; LGC Standards, Teddington, Middlesex, UK) following the procedure described by Levican et al. (2013), which was slightly modified as detailed below.

#### Cell culture

Caco-2 cell line was routinely grown in 75 cm^2^ tissue culture flasks (Corning Inc., New York, NY, USA) until a confluence of approximately 80% was reached in complete Minimum Essential Medium (cMEM), at 37 °C under a humidified atmosphere of 5% of CO_2_, using a Sanyo MCO-18AIC incubator. cMEM is composed of MEM 1X (Thermo Fisher Scientific, Waltham, MA, USA), 10% foetal bovine serum (FBS; Thermo Fisher Scientific, Waltham, MA, USA), 2 mM L-glutamine (Thermo Fisher Scientific, Waltham, MA, USA), 1% non-essential amino acids (NEAA; Sigma-Aldrich, St. Louis, Missouri, USA) and 1% penicillin/streptomycin solution (Sigma-Aldrich, St. Louis, Missouri, USA), and it was changed every 1–2 days.

For the adhesion and invasion assays, confluent Caco-2 monolayers were formed in two 24-well plates by adding 2 × 10^4^ cells mL^−1^ to each well and subsequently incubating for approximately 48 h under the above-mentioned conditions.

#### Preparation of bacterial suspensions

Overnight liquid cultures were diluted to an OD_600_ of 0.08 (approximately 10^9^ CFU mL^−1^) for *A. butzleri* strains and 0.05 (approximately 10^8^ CFU mL^−1^) for the control strains. Bacterial cells were subsequently harvested (900 × *g* for 5 min) and suspended in the same volume with tempered (37 °C) cMEM without penicillin and streptomycin.

#### Adhesion and invasion assays

Both 24-well plates with Caco-2 monolayers were infected with 0.5 mL of the previously prepared bacterial suspensions and then incubated at 37 °C under 5% of CO_2_ for 2 h. For adhesion assays, each well was then washed twice with 1X PBS (Thermo Fisher Scientific, Waltham, MA, USA), to remove unbound bacteria, and 0.5 mL of 1% Triton X-100 (CAS: 9036-19-5; Sigma-Aldrich, St. Louis, Missouri, USA) was added over ten min to lyse Caco-2 monolayers. The total number of Caco-2-associated bacteria was then calculated. For invasion assays, extracellular bacteria were killed by incubating the plate for 1 h with 0.5 mL of MEM 1X containing 125 µg mL^−1^ of gentamicin, cells were then washed and lysed as previously mentioned, and the number of intracellular bacteria was then calculated. The total number of cell-associated and intracellular bacteria was determined by plating the respective lysates on BHI agar plates (Oxoid, Basingstoke, UK) supplemented with 5% defibrinated sheep blood (Liofilchem, Roseto degli Abruzzi, Teramo, Italy). The number of adherent bacteria was calculated as the difference between the total number of bacteria associated with Caco-2 cells and the number of intracellular bacteria. Per experiment, each strain was studied in triplicate (three wells were inoculated) and the experiments were repeated on three independent occasions. Results were expressed as the mean number of bacteria (log_10_ CFU mL^−1^) that adhered or invaded ± standard deviation; and as the percentage of the original inoculum that adhered or invaded.

### WGS and bioinformatic analyses

Genomic DNA (gDNA) extraction from HC-1 and HC-2 was performed using the NucleoSpin^®^ Tissue kit (Macherey–Nagel, Düren, Nordrhein-Westfalen, Germany). The gDNA was quantified spectrophotometrically by Nanodrop^™^ 2000 (Thermo Fisher Scientific, Waltham, MA, USA). The WGS was performed by the General Services of the University of the Basque Country UPV/EHU (SGIker) on an Illumina MiSeq^™^ instrument (Illumina, Inc., California, USA). Libraries were prepared using the MiSeq library preparation kit (Illumina, Inc., San Diego, CA, United States), and paired-end sequencing was performed on an Illumina MiSeq instrument with a 150 bp paired-end protocol.

Trimming and quality control of all raw reads was performed using FastQC version 0.11.9 (http://www.bioinformatics.babraham.ac.uk/projects/fastqc/), and the trimmed reads were de novo assembled using SPAdes version 3.15.5 (https://github.com/ablab/spades) (Prjibelski et al. 2020). Afterwards, the quality of the obtained contigs was checked with QUAST version 5.2 (https://github.com/ablab/quast) (Gurevich et al. 2013), to obtain statistics related to the genome assembly processes and data quality, such as total genome length, number of contigs, GC content, coding sequence (CDS), tRNA, N50 and L50.

The species identity of the isolates was confirmed by WGS-based ribosomal multilocus sequence typing (rMLST) using the species identification tool of the pubMLST database (https://pubmlst.org/species-id) (Jolley et al. 2018). The multilocus sequence typing (MLST) profiles were obtained by querying the contigs against the *Arcobacter* typing tool of the same database (https://pubmlst.org/bigsdb?db=pubmlst_arcobacter_seqdef), which hosts the MLST scheme developed by Miller et al. (2009).

The presence of plasmid replicons was assessed by PlasmidFinder 2.1 (https://cge.food.dtu.dk/services/PlasmidFinder/) (Carattoli et al. 2014) with a minimum identity of 95% and minimum coverage of 60%.

Antimicrobial resistance (AMR) and virulence genes were detected using ABRicate version 1.0.1 (https://github.com/tseemann/abricate), by means of the default databases (Feldgarden et al. 2019; Gupta et al. 2014; Jia et al. 2017; Zankari et al. 2012) and two specific databases to identify AMR and virulence genes in *A. butzleri* (ARCO_IBIZ_AMR and ARCO_IBIZ_VIRULENCE, respectively) (Müller et al. 2020a). Gene sequences with an identity and coverage greater than 80% were considered present.

To detect other possible tetracycline resistance genes in addition to the *tetA* gene detected by ABRicate, an in silico PCR was carried out using the primers designed by Zambri et al. (2019) for the detection of *tetO* and *tetW* in *Arcobacter* spp. To look for mutations associated with quinolone resistance, the quinolone resistance determining region (QRDR) of the *gyrA* gene was amplified in silico using the PCR primers described by Abdelbaqi et al. (2007) and the sequences obtained were aligned and compared using ClustalW version 2.0 (https://www.genome.jp/tools-bin/clustalw) (Thompson et al. 1994). In addition, the 23S rRNA, *rpN* and *rplD* gene sequences were also extracted, aligned and compared to identify possible erythromycin-associated mutations.

The genome RM4018 was obtained from GenBank (accession number CP000361) and included in all the analyses as reference.

To investigate the relationship of HC-1 and HC-2 with other *A. butzteri* isolated from the same and different sources, a core-genome-based phylogenetic tree was created with IQTree v.2.2.2.7 (Minh et al. 2020) and visualised using iTOL (Letunic and Bork 2021). For this purpose, 181 complete genomes of *A. butzleri* and the associated meta-data were retrieved from GeneBank, and annotated, along with the genomes of HC-1 and HC-2, using Bakta v.1.8.2 (Schwengers et al. 2021). The pan-genome of the data set was assessed then using Panaroo v.1.3.3 (Tonkin-Hill et al. 2020), and the sequences of the identified core-genes were aligned using MAFFT (Katoh et al. 2009).

### Motility assay

The different flagellar gene content observed in the strains studied indicated possible differences in their motility, which was measured on semisolid thioglycolate medium. This medium was obtained after mixing fluid thioglycolate medium (Scharlau, Sentmenat, Barcelona, Spain) with bacteriological agar (Scharlau, Sentmenat, Barcelona, Spain) at a concentration of 0.4%. The solidified plates were pricked with a single bacterial colony using a sterile pipette tip. The measurement of the halos produced by the motility of the bacteria was made after 24 h of incubation at 30 °C. This assay was performed in triplicate in three independent experiments.

### Urease test

The urease test was performed to corroborate the possible differences in the urease activity of the strains, indicated by their different urease gene content. Three–five isolated colonies of each *A. butzleri* strain were streaked onto the surface of Christensen urea agar slant (BD BBL^™^, Franklin Lakes, New Jersey, USA), and the tubes were then incubated for 72 h at 30 °C under aerobic conditions. The change in medium colour from orange to pink was considered as a positive result. The strains *A. butzleri* RM4018 and *E. coli* DH5α were included as positive and negative controls, respectively. The assay was performed in triplicate at least on three independent experiments.

### Statistical analysis

Statistical analyses were performed with the SPSS Statistics 26 software (SPSS Inc., Chicago, 221 IL, USA). The statistical Mann–Whitney U-test was used to compare the results obtained in adhesion and invasion assays. Significance was established at *p* < 0.01.

## Results

### Bacterial identification

The stool cultures allowed the isolation of two bacterial strains, one derived from each patient, that grew pure and heavily on CIN agar as mannitol negative colonies (uncoloured). They were identified as *A. butzleri* by MALDI-TOF MS [log(score) ≥ 2.0] and named, respectively, HC-1 and HC-2. No other bacterial pathogens were detected in any of the samples and all tests performed for the detection of viruses and parasites were negative. The WGS-based rMLST confirmed the identification, with 100% support. Adhesion and invasion assays in human intestinal Caco-2 cells.

The results obtained in the adhesion and invasion assays are shown in Table 1. In general, significant differences (*p* < 0.001) were detected between the two *A. butzleri* isolates tested. While HC-1 was able to strongly adhere to Caco-2 cells, as well as to invade them with a good ability, HC-2 was neither able to adhere nor invade them.

**Table 1:** Adherence and invasion capability of the tested isolates

Strains	Adhesion		Invasion	
	log_10_ CFU mL^−1a^	%	log_10_ CFU mL^−1a^	%
*Salmonella enterica* serovar Typhimurium LT2	6.96 ± 0.03	87.00	5.22 ± 0.00	65.25
*Escherichia coli* DH5α	6.39 ± 0.02	79.87	NI^b^	NI^b^
*Arcobacter butzleri* RM4018	5.65 ± 0.03	70.63	4.47 ± 0.01	55.88
*A. butzleri* HC-1	6.78 ± 0.02*	84.75*	4.06 ± 0.09*	50.75*
*A. butzleri* HC-2	3.99 ± 0.07	49.87	1.26 ± 0.02	15.75

^a^The values for adhesion and invasion are expressed as means ± standard errors and were proportionally calculated to an inoculum of 10^8^ CFU mL^−1^ (8.0 log_10_ CFU mL^−1^) for each strain

^*b*^NI, no invasion

^*^Statistically significant differences (*p* < 0.001) between adherence and invasion capabilities of HC-1 and HC-2

### Antimicrobial susceptibility

Antimicrobial susceptibility tests revealed that both HC-1 and HC-2 were resistant to tetracycline. In addition, HC-2 also showed resistance to amoxicillin–clavulanic acid and ampicillin. Table 2 shows the antimicrobial susceptibility profiles of the strains, along with the associated AMR genetic determinants detected.

**Table 2:** Antimicrobial susceptibility profiles and associated genetic AMR determinants of HC-1 and HC-2

Antimicrobial^a^	Strains			
	HC-1		HC-2	
	Phenotype^b^	Genetic markers	Phenotype^b^	Genetic markers
AMC	S (1.5 mg/L)	*bla2* (ABU_RS06485), *hcpC* (ABU_RS02795), EP11, EP15, EP16^c^	R (32 mg/L)	*bla2* (ABU_RS06485), ***bla3* (ABU_RS07375)**, *hcpC* (ABU_RS02795), EP11, EP15, EP16^c^
AMP	S (6 mg/L)		R (32 mg/L)	
GM	S (0.094 mg/L)	–	S (0.075 mg/L)	–
TET	R (4 mg/L)	***tetO***	R (4 mg/L)	*tetA* (ABU_RS01680), ***tetO***
ERY	S (4 mg/L)	–	S (4 mg/L)	–
CIP	S (0.023 mg/L)	Ser-97-Asn^d^	S (0.094 mg/L)	Ser-97-Asn^d^

Genetic markers potentially associated with the observed resistance are in bold

^a^AMC, amoxicillin–clavulanic acid; AMP, ampicillin; GM, gentamicin; TET, tetracycline; ERY, erythromycin; CIP, ciprofloxacin

^b^S, susceptible; R, resistant. MICs are given in brackets

^c^Genes and EPs associated with β-lactams resistance (*bla2*, ABU_RS06485; *hcpC*, ABU_RS02795; EP11; EP15; EP16) present in the genome that may confer resistance to other β-lactams not tested here

^d^Aminoacid substitution in QRDR of *gyrA* gene correlated with susceptible phenotype (Ferreira et al. 2018)

### Genome assembly, quality control, MLST and plasmid detection

The raw reads and contig files passed the defined thresholds for FastQC and QUAST, respectively. For HC-1, 549,999 reads and 62 contigs were yielded; the genome length was 2,274,951 bp; the N50 was 145937; and L50, 6. For HC-2, 945,822 reads and 57 contigs were yielded; the genome length was 2,239,076 bp; the N50 was 142495; and L50, 5. Table 3 summarises metrics of SPAdes assemblies produced by QUAST. This Whole Genome Shotgun project has been deposited at DDBJ/ENA/GenBank under the accessions JAVMBX000000000 (HC-1) and JAVMBY000000000 (HC-2). The versions described in this paper are version JAVMBX010000000 and JAVMBY010000000. The assembled contig file of each strain is also available in the PubMLST (https://pubmlst.org/bigsdb?db=pubmlst_arcobacter_isolates&page=query&genomes=1) under the ID numbers 1014 and 1015.

**Table 3:** Summary of the QUAST software results for genomes HC-1 and HC-2, as well as for the reference genome RM4018

Assembly	HC-1	HC-2	RM4018
Total length (Mbp)	2.27	2.24	2.34
Number of contigs	62	57	1
GC content (%)	27.00	26.86	27.05
CDS	2254	2202	2261
tRNA	46	51	54
N50	145937	142495	–
L50	6	5	–

Both isolates were successfully typed by MLST (Table 4). Two previously unreported sequence types (ST) were identified, namely, ST 832 and ST 833, that resulted, respectively, from a new *glyA* allele sequence and from a new combination of the identified MLST alleles. No plasmids were detected in any of the isolates.

**Table 4:** Multilocus sequence typing of strains HC-1 and HC-2, as well as for the reference genome RM4018

Strain	*aspA*	*atpA*	*glnA*	*gltA*	*glyA*	*pgm*	*tkt*	ST
HC-1	20	34	9	5	778^a^	239	2	832^a^
HC-2	39	7	19	2	53	52	31	833^a^
RM4018	1	1	1	1	1	1	1	1

^a^New allele and STs

### AMR determinants

The in-depth analysis of the sequencing data allowed, by analysing both genomes together, the detection of 78 AMR determinants that included 47 genes related to 18 efflux pump (EP) systems and other 27 genes, here referred to as other AMR determinants. (Table S1).

Some differences were observed between HC-1 and HC-2. Both genomes presented all the genes associated with the production of 14 EP systems (EP2-6, EP8-10, EP11 and EP13-17), but those related to EP7, EP12 and EP19 were only detected in HC-2. Moreover, three of the four genes that make up EP1 were only detected in HC-1. In addition, both genomes carried the outer membrane protein gene *tolC*, and HC-2 carried the transcriptional regulator genes *kstR2* and *ohrR*.

Regarding the other AMR determinants, both genomes carried *bla2* (MBL fold metallo-hydrolase); *hcpC* (putative β-lactamase); the penicillin binding protein genes *mrdA*, *pbpB* and *pbpF; arnB* (UDP-4-amino-4-deoxy-L-arabinose-oxoglutarate aminotransferase); *eptA* (phosphoethanolamine transferase), *tetO* (tetracycline resistance protein); the antibiotic resistance protein gene ABU_RS04955; *rmlN* (putative dual specificity RNA methyltransferase); the TolC outer membrane protein gene *oprF3*; several putative multidrug export ATP-binding/permease protein genes (ABU_RS02345, ABU_RS05540, *macB1*, *ybiT1, ybiT2* and *ylmA*); and *acrB* (multidrug EP subunit). Three more genes were detected in HC-1, namely *aph(3')-IIIa* (aminoglycoside O-phosphotransferase), *wbpD* (UDP-2-acetamido-3-amino-2,3-dideoxy-D-glucuronate N-acetyltransferase), and *sat4* (streptothricin N-acetyltransferase); and six in HC-2, namely *bla3* (β-lactamase OXA-15) *sttH* (cysteine hydrolase), the tetracycline resistance protein genes *tetA* and *tetO*, *hipA1*(serine/threonine-protein kinase), and ABU_RS01690 (putative multidrug export ATP-binding/permease protein). None of the strains presented resistance-associated point mutations in the QRDR of the *gyrA* gene, nor in the *rplD*, *rplV* or 23S rRNA. However, both strains showed the G169A mutation in the QRDR, leading to the amino acid exchange from serine to asparagine (Ser-97-Asn).

### Virulence determinants

Taking into account the two genomes analysed, a total of 77 virulence determinants were identified, including 11 genes in the lipid A cluster, eight related to the chemotaxis system, six to the urease cluster, 36 flagellar genes and 16 other genes related to virulence (Table S2). There were no major differences in the gene content of chemotaxis and lipid A clusters between HC-1 and HC-2. The complete chemotaxis cluster was detected in both isolates; and for the lipid A cluster, eight of the eleven genes were detected in HC-1 (all except *phoQ, phoP1 and phoP2*) and all but *phoQ* in HC-2. However, differences in the number of flagellar and urease genes were observed between strains. Although HC-2 presented the entire flagella cluster, HC-1 lacked eight genes; and while HC-1 presented the urease cluster, HC-2 did not. The other virulence determinants detected in both strains were the same.

### Phylogenetic analysis

**Fig. 1:**
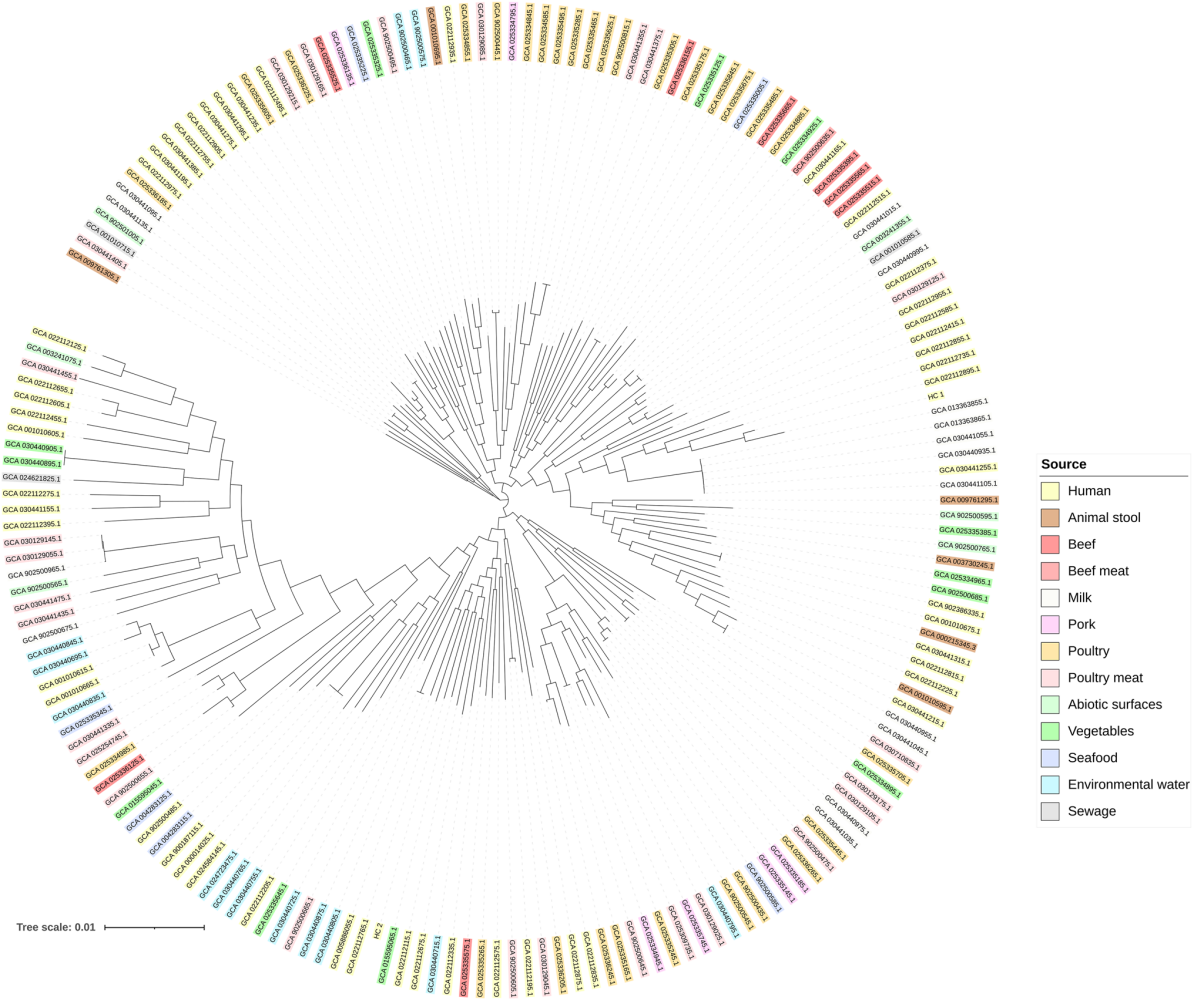
Phylogenetic tree of 183 *A. butzleri* strains (HC-1 and HC-2 included) based on core-genome genes. The colour code represents the isolation source of each strain

The analysis of the relatedness of the genomes of HC-1, HC-2 and other *A. butzleri* available in the GeneBank database revealed considerable divergence between HC-1 and HC-2. Both clustered with strains of human origin but appeared in phylogenetically distant clades (Fig. 1).

### Urease activity

**Fig. 2:**
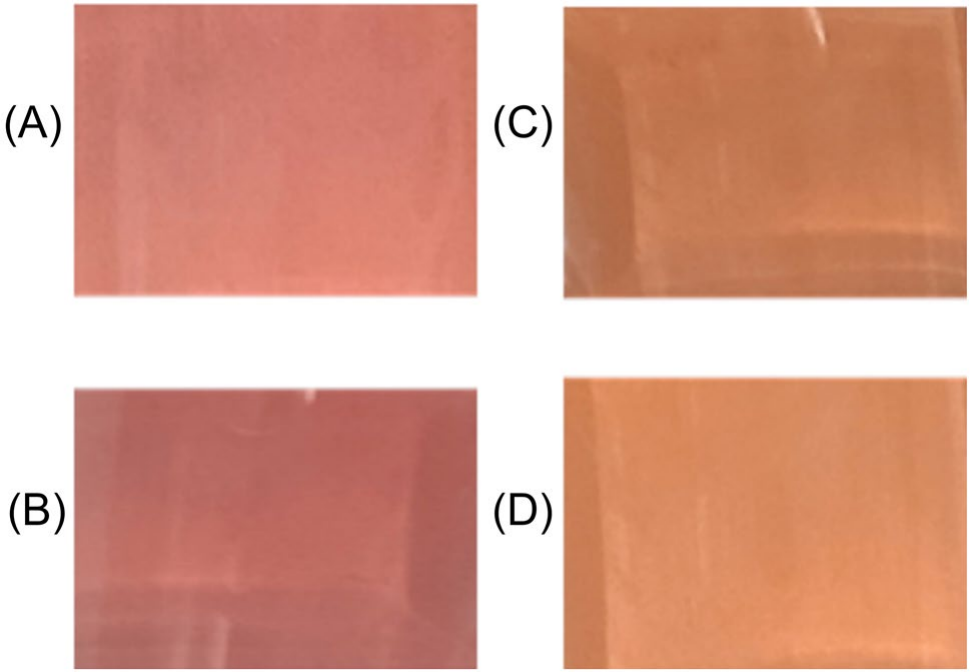
Representative assay of the Christensen urea agar urease activity test. Urease activity was detected when the colour of the medium turned to red/pink in RM4018 (**A**) and HC-1 (**B**). No urease activity was detected when the colour of the medium remained yellow/orange in *E. coli* DH5α (**C**) and HC-2 (**D**). The figure represents the colour change obtained in the test medium employed in the assay

Based on the colour acquired by the Christensen Urea Agar after 48 h of growth, the strain HC-1 turned the medium pink (urease positive), while HC-2 did not (urease negative) (Fig. 2).

### Motility

**Fig. 3:**
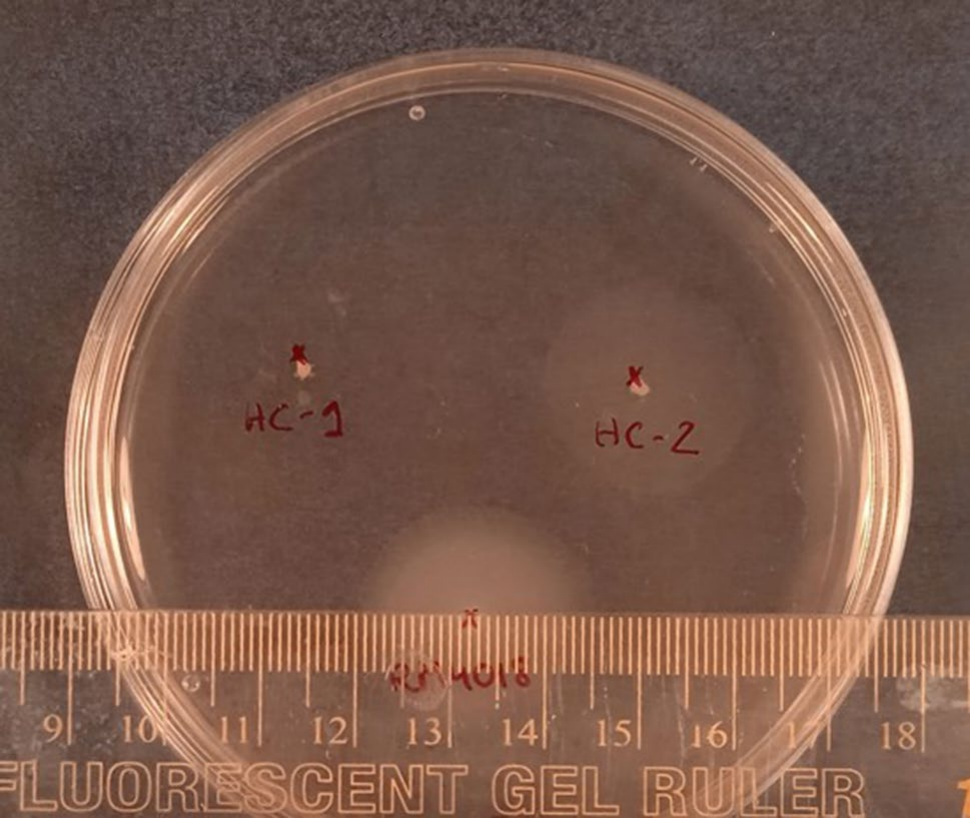
Representative image obtained from the motility assay. Strains: RM4018 (control strain; down), HC-1 (up, left) and HC-2 (up, right) in thioglycolate medium

The motility assays showed a significant difference (*p* < 0.001) between both strains: while HC-1 showed a motility halo diameter of 0.00 ± 0.01 cm, the halo diameter of strain HC-2 was 2.00 ± 0.02 cm. A representative image of the motility of the strains is shown in Fig. 3.

## Discussion

Despite being a human enteric pathogen, the detection and isolation methods for *A. butzleri* are not standardised and that is why the presence of this bacterium in stool samples from patients with enteritis is not routinely investigated (Ruiz de Alegría Puig et al. 2023). Additionally, the mechanisms underlying the pathologies associated with its infection remain unclear (Chieffi et al. 2020). Here, we aimed to provide information on the virulence and AMR determinants of two *Arcobacter* strains, HC-1 and HC-2, isolated by stool culture from a patient suffering from traveller’s diarrhoea and from another diagnosed with pruritus.

HC-1 and HC-2 were selected on CIN agar, on which they grew in pure culture forming colourless colonies. CIN is a selective and differential medium originally developed for the isolation of *Yersinia enterocolitica* from stool samples that is currently also used for the isolation of *Aeromonas* and other *Yersinia* species. In either case, the ability to ferment mannitol in the presence of neutral red gives rise to reddish coloured colonies. Although the growth of mannitol negative colonies is routinely ignored, in the two cases described here the growth was so exceptionally heavy that it prompted us to identify them using MALDI-TOF MS, which was decisive for the identification of *A. butzleri* in both cases. Therefore, our results may indicate that for the correct diagnosis of *Arcobacter* spp., mannitol-negative colonies on CIN agar should be considered. In fact, the formation of colourless colonies on CIN agar by *A. butzleri* isolates initially isolated on CHROMID^®^ ESBL (bioMerieux, Marcy-l'Étoile, France) has recently been reported (Jiménez-Guerra et al. 2020).

*A. butzleri* enteritis may be self-limiting and does not usually require treatment, but in exceptional cases, i.e., those in which the symptomatology is prolonged or aggravated, therapy with quinolones, tetracycline, or aminoglycosides could be recommended (Pérez-Cataluña et al. 2017). Therefore, HC-1 and HC-2 were tested in the hospital where they were isolated against six antimicrobials. HC-1 was resistant to tetracycline and HC-2 to tetracycline, amoxicillin–clavulanic acid and ampicillin. Some of the genetic AMR determinants identified in both strains supported the observed resistance phenotypes. In fact, genomic analyses not only revealed the mechanisms likely responsible for those phenotypes but also uncovered AMR mechanisms potentially involved in other resistances not studied here.

Most of the 19 different EP systems identified in *A. butzleri* to date (Isidro et al. 2020) were detected in HC-1 and HC-2. Those most frequently reported were detected in both, and were: (1) EP2, comprising only *yajR* and which being overexpressed can confer multidrug resistance in *E. coli* (Nishino et al. 2001); (2) EP3, consisting of four genes including *macA1* and *macB2*, which encode macrolide export proteins in *A. butzleri* (Fanelli et al. 2019); (3) EP4, composed of four genes that include *bepD* and *bepE*, responsible for multidrug resistance in *Brucella suis* (Martin et al. 2009); (4) EP5 or *msbA*, coding for a protein that is related to the family of multidrug resistance proteins (Karow et al. 1993); (5) EP6 or *uup*, involved in the formation of a soluble ATP-binding cassette (ABC) ATPase that has been implicated in several processes different from transmembrane transport of molecules, such as excision of transposable elements and the deletion of single copies of tandem chromosomal repeats in *E. coli* (Burgos Zepeda et al. 2010); (6) EP8 or *sugE*, encoding a transporter from the small multidrug resistance (SMR) family that also provides resistance to narrow quaternary ammonium compounds in *E. coli* and *Enterobacter cloacae* (Chung et al. 2002; He et al. 2011); (7) EP9 or *fsr,* conferring resistance to fosmidomycin in *A. butzleri* (Fanelli et al. 2020); (8) EP10, made up of five genes including the macrolide export protein coding *macA3* (Fanelli et al. 2019); (9) EP11; (10) EP15; and (11) EP16, all of which comprise several genes including *mexB1* (EP11), *mexA1*, *mexB2* (EP15), and *mexA2* (EP16), allegedly involved in the formation of the MexAB–OprM system responsible for resistance to quinolones, macrolides, novobiocin, chloramphenicol, tetracycline, lincomycin and β-lactams in *Pseudomonas aeruginosa* (Li et al. 1995; Masuda et al. 2000); (12) EP13 or *ydhP*, that encodes an inner membrane transport protein; and (13) EP14 or *bcr1* and (14) EP17 or *bcr2*, involved in the resistance to sulphonamides and bicyclomycin in *E. coli* (Nichols et al. 1989) and, as far as we know, only to bicyclomycin in *A. butzleri* (Chieffi et al. 2020). Studies that have investigated the prevalence of these EP systems in collections of at least 40 strains (Isidro et al. 2020; Müller et al. 2020b; Uljanovas et al. 2023) show that their prevalence is generally close to or above 90%, except for EP16 and EP17, which in two studies were detected at rates of less than 70% (Isidro et al. 2020; Uljanovas et al. 2023). Based on the three studies cited, the least prevalent systems are EP18 and EP19 (around 2%), followed by EP1 (12–38.8%) and EP7 (21.3–53.1%). Here, in line with what has been reported so far, EP18 was not detected in any strain; only three of the genes that compose EP1 were detected in HC-1; and EP7and EP19 were only detected in HC-2. Additionally, of the two strains HC-2 was the only in which EP12 was detected, a system for which 100% prevalence has been reported to date (Isidro et al. 2020; Müller et al. 2020b; Uljanovas et al. 2023). Among the genes that make up the three EP systems only detected in HC-2 were *mdtA* and *mdtB2,* which could contribute to the formation of the MdtABC-TolC complex, a multidrug efflux system of Gram-negative bacteria, including *E. coli* and *Salmonella,* that confers resistance to novobiocin, deoxycholate and β-lactam antibiotics (Pletzer et al. 2014); and *mdtE*, implicated in the formation of the MdtEF complex that could provide resistance to β-lactams and erythromycin in *E. coli* (Nishino et al. 2008). If functional, and provided that they confer similar resistance in *A. butzleri* to that conferred in other bacterial species, EP11, EP15 and EP16 could be responsible for the resistance to tetracycline shown by HC-1 and HC-2; and EP7 and EP12 for the resistance to ampicillin in HC-2. However, in the same assumption, EP3 and EP10 could confer resistance in both strains against erythromycin, which was not reflected in our antimicrobial susceptibility testing (AST). This discrepancy between genotype and phenotype has previously been reported for an *A. butzleri* isolate (Jehanne et al. 2022; Müller et al. 2020a, b; Uljanovas et al. 2023).

In relation to the other AMR determinants detected, some of those related to polymyxin resistance (i.e. *arnB*, *eptA*), tetracycline resistance (i.e. *tetO*) and β-lactam resistance (i.e. *bla2*, *hcpC, mrdA*, *pbpB*, *pbpF*) were present in both isolates. Previous studies point to a 100% prevalence for all of them (Fanelli et al. 2020; Müller et al. 2020a, b; Uljanovas et al. 2023) except for *tetO*, which is less frequently detected. Although three of the most recent studies carried out with *A. butzleri* did not detect it in any of the strains tested (Jehanne et al. 2022; Müller et al. 2020b; Uljanovas et al. 2023), those conducted by Sciortino et al. (2021) and Lameei et al. (2022) detected it in all tetracycline resistant strains examined. Here, the presence of the *tetO* gene in both genomes is probably the reason for resistance to tetracycline, as this genotype–phenotype correlation has previously been reported (Lameei et al. 2022; Sciortino et al. 2021; Zambri et al. 2019). However, HC-2 also contained *tetA*, which, as reported elsewhere (Sciortino et al. 2021) could also contribute to tetracycline resistance in this strain. The detection of this gene varies considerably between studies, ranging from 49.3 to 60% in the two studies in which the highest number of *A. butzleri* strains have been analysed (Müller et al. 2020b; Uljanovas et al. 2023). Other AMR determinants that were only detected in HC-2 were:, which confers resistance in *A. butzleri* against ampicillin and amoxicillin–clavulanic acid (Isidro et al. 2020; Jehanne et al. 2022); *hipA1*, encoding a serine/threonine-protein kinase, part of a type II toxin–antitoxin system, involved in multidrug resistance also related to AMR in *Arcobacter* (Fanelli et al. 2019; Miller et al. 2007); *relE*, also a component of a type II toxin–antitoxin system and associated with the development of persistent resistance to ofloxacin, cefotaxime and tobramycin (Lewis 2005); ABU_RS01690, a putative multidrug export ATP-binding/permease (Müller et al. 2020a); and *sttH*, conferring resistance against streptothricin and other structurally related naturally occurring cyclic amide compounds (Fanelli et al. 2019). In view of the results obtained here, the OXA-15 β-lactamase gene *bla3* would be responsible for the resistance of HC-2 to ampicillin and amoxicillin–clavulanic acid; since the rest of the AMR determinants associated with resistance to beta-lactams identified here were detected in both strains, and only HC-2 showed resistance to those antimicrobials. In turn, HC-1 also showed exclusive AMR determinants: *aph(3’)-IIIa*, which confers resistance to kanamycin in *Campylobacter* (Liao et al. 2022) and which, regardless of the apparent absence of plasmids in our strain, has recently been reported for the first time in a plasmid-bearing strain of *A. butzleri* (Zautner et al. 2023); *sat4*, conferring resistance to streptothricin in *Campylobacter coli* and *A. butzleri* (Bischoff et al. 1996; Müller 2020a); and *wbpD*, which provides resistance to chloramphenicol in *A. butzleri* (Fanelli et al. 2019). The prevalence of these genes detected in only one of the two strains analysed varies among studies, in no case exceeding a detection rate of 60% (Müller et al. 2020b; Uljanovas et al. 2023). None of the two isolates harboured *cat3*, a chloramphenicol acetyltransferase, nor any of the mutations in the 23S rRNA, *rplD,* or *rplV* genes that could confer resistance to erythromycin. No quinolone resistance-associated mutations in the QRDR of the *gyrA* gene were present either. However, both strains presented a mutation leading to the amino-acid substitution (Ser-97-Asn) in position 97 of the QRDR that had been previously observed in ciprofloxacin susceptible isolates (Ferreira et al. 2018). The lack of resistance determinants against erythromycin and ciprofloxacin in HC-1 and HC-2 justifies the observed susceptible phenotypes for these agents.

In addition to identifying the specific AMR determinants of each of the studied strains, the genetic study allowed the identification of their virulence determinants, which could be grouped into different clusters: lipid A, chemotaxis, urease, flagellum and other virulence determinants.

The lipid A, responsible for the higher endotoxic activity, is a component of the lipopolysaccharides found in the external membrane of Gram-negative bacteria (Oliveira et al. 2022). Both strains presented almost the entire cluster, with all eight genes required for lipid A biosynthesis (*lpxA*, *lpxB*, *lpxC*, *lpxD*, *lpxH*, *lpxK*, *lpxP* and *waaA*) but without any of those encoding the PhoPQ two-component regulatory system (*phoP1-P3* and *phoQ*). Specifically, the genes *phoP1* and *phoP2* were missing in HC-1 and *phoQ* was absent in both strains. In line with that previously reported by Müller et al. (2020a), who also identified two *A. butzleri* strains that were missing regulatory genes of this cluster, our results suggest that the PhoPQ regulatory system would not be functional in HC-1 and HC-2, being therefore unable to modify their lipid A structure.

In relation to those genes associated with chemotaxis and urease activity, both isolates presented a complete chemotaxis cluster composed of eight *che* genes (*A*, *B*, *R*, *V*, *W* and *Y1–Y3*), but only strain HC-1 harboured the complete urease cluster (*ureA-G*). The detection of both clusters in *A. butzleri* had previously been reported elsewhere (Isidro et al. 2020; Miller et al. 2007; Müller et al. 2020a, b). To determine whether the urease gene cluster conferred specific activity to HC-1, both strains were subjected to the Christensen Urea Agar assay, that differentiates urease producing strains on the basis of their ability to hydrolyse the urea present in the medium to ammonia, thereby increasing the pH and turning its colour from orange to pink. The urease-positive phenotype of strain HC-1 was supported by its genotype, while the absence of urease gene cluster in HC-2 correlated with its urease negative phenotype. Isidro et al. (2020) and Müller et al. (2020a, b) also confirmed the urease positive phenotype in almost all urease cluster positive strains of *A. butzleri* analysed. Therefore, it may be of great interest to determine whether urease activity is present in *Arcobacter* strains that cause intestinal pathology, as this phenotypic trait could be advantageous for them by allowing their survival under the acidic conditions of the stomach and reaching the intestine in greater numbers.

Regarding flagellar genes, HC-1 did not present eight of the 36 genes that make up this cluster, while in HC-2 all were detected. Those genes absent in HC-1 were: *flaA* and *flaB,* encoding the filament flagellins A and B (Ho et al. 2008); *flgD*, required for flagellar hook formation (Matsunami et al. 2021); *flgL*, coding for the FlgL protein that forms the junction between the hook and the filament (Hong et al. 2018); *flhA* and *flhB2*, indispensable genes for the formation of the rod; *fliI*, which encodes the FliI ATPase required for the secretion of the major pilus subunit, PilA; and *fliS*, which is essential for filament assembly and can also facilitate flagellin secretion (Radomska et al. 2017). These results suggested HC-1 to be a non-flagellated strain, or to present a defective flagellum with a missing hook and filament. We did not prove by microscopy the absence of the flagellum, but we did test the motility of both strains. The results obtained revealed that, unlike HC-2, HC-1 was unable to move. Apart from cell motility and chemotaxis, bacterial flagella are also implied in cell colonisation and invasion procedures in various pathogens. However, as evidenced by the ability of HC-1 to successfully infect Caco-2 cells, as well as the inability of HC-2 to do so, motility is not necessary for *Arcobacter* to adhere to and invade Caco-2 cells, at least at 2 h of infection. Similar results were obtained with non-motile and/or non-flagellated *Salmonella* Enteritidis mutants (van Asten et al. 2004). Therefore, in line with that reported for some other intestinal pathogens (Josenhans et al. 2002), motility does not appear to be the only key factor in causing intestinal infection.

Several extrinsic and intrinsic factors may be involved in causing infection. Indeed, our results show that the infectivity of *A. buztleri* can differ notably (*p* < 0.001) between strains, which is in line with the previously reported variable adhesive and invasive capabilities among *A. butzleri* isolates from human origin (Buzzanca et al. 2021; Ferreira et al. 2014; Levican et al. 2013; Karadas et al. 2013). In addition, our results point toward strain-specific pathogenic mechanisms since the woman infected with HC-1 (able to adhere to and to invade Caco-2 cells) presented diarrhoea, and the man infected with HC-2 (not able to adhere nor to invade) did not. However, in both strains were identified the same 16 virulence-related genes, which were associated to adhesion (*cadF*, *cj1349*)*,* invasion* (ciaB*, *iamA)*, erythrocyte lysis (*tlyA*, *pldA),* quorum sensing (*luxS*), metal transport (*cirA2*, *fur*), biosynthesis of peptidoglycan (*mviN*) and lipopolysaccharide core (*waaC*, *waaF*), adhesin folding (*htrA*), and virulence regulation (*cvfB*, *virF*, *voc*). They are probably behind the adherent and invasive capabilities of HC-1, but these results did not provide us with relevant information to justify why this strain was able to adhere to and invade Caco-2 cells while HC-2 was not. The phylogenetic analysis did not provide information in this regard either, as despite appearing in separate clusters, both HC-1 and HC-2 were phylogenetically close to other *A. butzleri* strains derived from humans with gastroenteric symptoms (all the isolates from human origin retrieved from GeneBank were associated with gastroenteritis). It is worth noting that the non-detection of some virulence determinants by WGS does not strictly imply the absence of those genes. For example, it would be advisable to take into consideration, in future studies, the extreme polymorphism of the *hecA* gene (presumably associated with adherence) and to confirm its absence or presence, as did Isidro et al. (2020). Probably, besides the virulence-related genes detected here, some other genes and/or factors (i.e. different conditions established in the in vitro assays) may be related to the adherent and invasive capabilities of HC-1 and HC-2. Whatever the case, it is highly probable that the isolation and identification of *A. butzleri* from the patient with pruritus was coincidental, as, to our knowledge, *Arcobacter* has never been reported as the causative agent of pruritus and the assays used here were not designed to obtain conclusive data in this regard. In fact, our results could support the presence of *A. butzleri* as part of colonizing microbiota in this patient with no gastroenteric symptoms, as previously reported elsewhere (Jiménez-Guerra et al. 2020). However, it would be of great interest to clarify, through further studies, whether *A. butzleri* could be responsible for pruritus or not.

In conclusion, this study shows that the infectivity of *A. butzleri* isolates from human stool can vary depending on their genetic characteristics. These strain-specific properties can condition the pathology of the bacterial infection, since the adherent and invasive strain HC-1 was the only pathogen identified as the cause of the traveller’s diarrhoea in the patient, while the non-adherent and non-invasive HC-2 was isolated from a patient without enteric symptoms. This study also shows that flagellar motility does not condition the infectivity of *A. butzleri* and highlights the importance of the identification and characterisation of multiple comprehensive intrinsic and extrinsic factors that may condition the virulence of this pathogen. In this regard, despite the observed correlation between AMR, motility and urease genotype and phenotype, the virulence determinants identified for both strains do not support the phenotypic differences observed between them in Caco-2 cells. In the same line, it would be of great interest to confirm all the genetic traits of AMR by means of AST for other antibiotics not tested here. Finally, based on the procedure that allowed us to select the strains studied here, mannitol negative colonies on CIN agar should be taken into consideration in microbiology laboratories in order to improve the diagnostic procedure for *Arcobacter* infections.

### Supplementary Information

Below is the link to the electronic supplementary material.

Supplementary file1 (XLSX 38 KB)

## Data Availability

This Whole Genome Shotgun project has been deposited at DDBJ/ENA/GenBank under the accessions JAVMBX000000000 and JAVMBY000000000. The versions described in this paper are version JAVMBX010000000 and JAVMBY010000000. The assembled contig-file of each strain is also available online in the PubMLST web, (https://pubmlst.or/bigsdb?db=pubmlst_arcobacter_isolates&page=query&genomes=1) under the ID numbers 1014 and 1015.
